# Effect of substrate mineralogy, biofilm and extracellular polymeric substances on bacterially induced carbonate mineralisation investigated with in situ nanoscale ToF-SIMS

**DOI:** 10.1038/s41598-025-14083-z

**Published:** 2025-08-11

**Authors:** Anant Aishwarya Dubey, Pelina Toprak, Allan Pring, Carlos Rodriguez-Navarro, Abhijit Mukherjee, Navdeep K. Dhami

**Affiliations:** 1 School of Civil and Mechanical Engineering, Curtin University, Perth, Australia; 2 School of Physics, Chemistry and Earth Sciences, University of Adelaide, Adelaide, Australia; 3 Department of Mineralogy and Petrology, University of Granada, Granada, Spain; 4 School of Molecular and Life Sciences, Curtin University, Perth, Australia

**Keywords:** *Biomineralisation*, *Calcium carbonate polymorph selection*, *Extracellular polymeric substances (EPS)*, *Substrate influence on biomineralisation*, *EPS-Mineral interactions*, Biogeochemistry, Civil engineering, Biomaterials, Environmental microbiology, Soil microbiology, Mass spectrometry, Microscopy

## Abstract

Bacterial mineralisation of calcium carbonates (CaCO_3_) has become a focal point of interest in the scientific community owing to their versatile applications as biomaterials. However, despite extensive research, the knowledge on factors influencing biogenic CaCO_3_ polymorph (calcite, vaterite or aragonite) selection in nature remains obscure. Bacterial mineralisation happens in nature on diverse substrates by different pathways, often in the presence of organic matter such as biofilm and extracellular polymeric substances (EPS) secreted with the regular metabolic activities of microbes. This study examines the bacterial CaCO_3_ mineralisation process by two distinct pathways on different natural substrates with advanced analytical techniques, including Time of Flight- Secondary Ions Mass Spectrometry (ToF-SIMS). A high EPS-producing microbe (*Bacillus subtilis*, BS) was compared with the standard ureolytic strain (*Sporosarcina pasteurii*, SP). Natural geological minerals, including apatite, calcite and quartz, were selected as substrates. This study demonstrates that SP favours the precipitation of rhombohedral calcite crystals (2 to 40 μm in size), regardless of the mineral substrate. In contrast, the EPS-producing BS culture induced the formation of significantly larger vaterite structures (20 to 100 μm in size) in spheroid and hexagonal shapes. The mineralogy of precipitates was confirmed with Raman spectroscopy. ToF-SIMS enabled the spatial tracking of organic macromolecules and the adsorption of calcium ions on them. The functional groups of the EPS involved in these interactions were characterised by Fourier Transform Infrared Spectroscopy (FTIR). This study reveals that microbial activity dominates over substrate mineralogy in selecting the phase and shaping the morphology of biogenic CaCO_3_, with EPS playing a crucial role in promoting the aggregation of small nanocrystals into large vaterite structures and their stabilisation.

**Supplementary Information:**

The online version contains supplementary material available at 10.1038/s41598-025-14083-z.

## Introduction

 Biomineralisation is a ubiquitous biogeochemical process in nature, often highlighted in the form of natural structures such as stromatolites, microbialites in coral reefs, beach rocks, microbial fossils, micrite cement, and many more^1–4^. Bacterial biomineralisation has gained the focus of the scientific and engineering community for its boundless application potential, ranging from advanced medical biomaterials such as self-healing dental composites to civil and geological engineering applications such as self-healing concrete, contaminated land remediation and soil erosion control^5–9^. Most of these studies focussing on bacterial biomineralisation have considered different pathways of calcium carbonate precipitation, including urea hydrolysis, deamination of proteins, nitrate and sulphate reduction, and photosynthesis, with or without the aid of enzymes such as carbonic anhydrase and urease, resulting in the ubiquitous occurrence in nature of different CaCO_3_ polymorphs (i.e., calcite, aragonite and vaterite), plus hydrated calcium carbonate phases such as monohydrocalcite and amorphous calcium carbonate (ACC)^10–14^. Around 50% of known biominerals in nature are calcium-based, as bacterial cells prefer them for crucial cellular metabolism^15^.

**Fig. 1:**
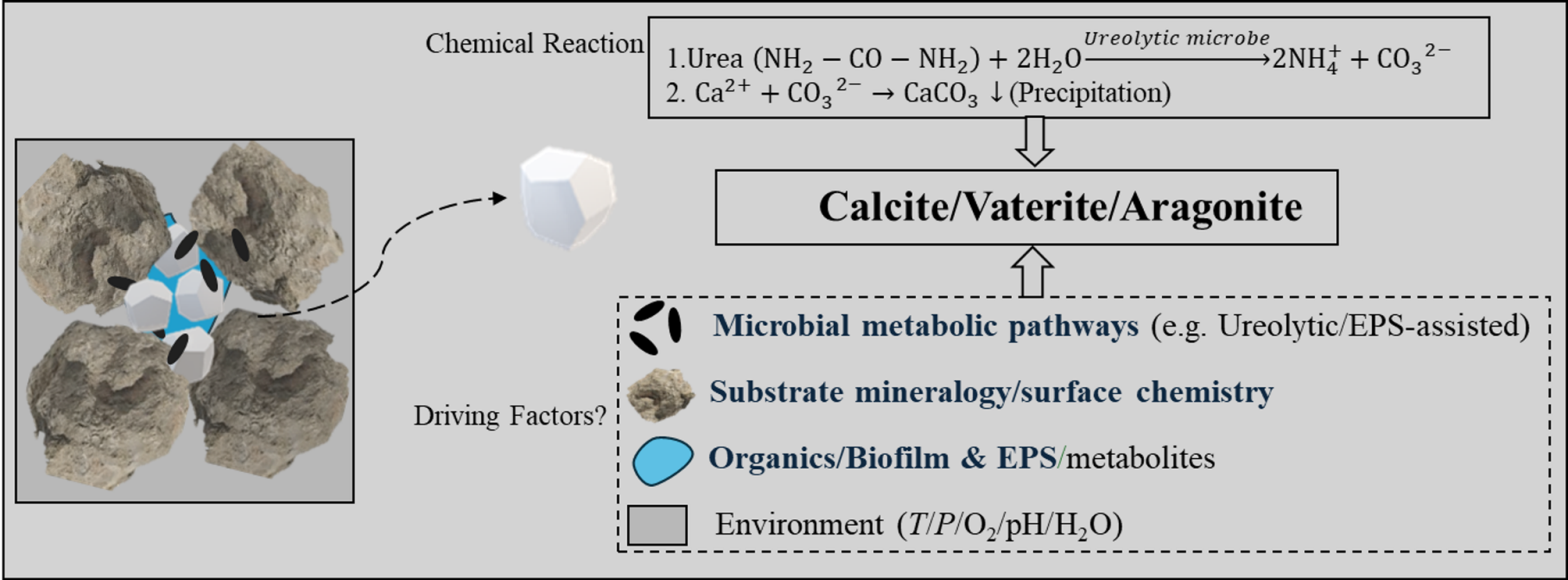
Factors influencing biogenic CaCO_3_ polymorph selection.

Among the various pathways of bacterial CaCO_3_ biomineralisation, the urea hydrolysis route has become the most popular due to its relatively fast precipitation rates and easy availability of reagent salts in nature^1,16^. In soils and marine environments, over 5,000 bacterial species are recorded to be capable of biomineralisation through the urea hydrolysis pathway^17^. This pathway involves bacterial urease that breaks down urea to ammonia and CO_2,_ which rapidly converts into carbonate ions in the alkaline micro-environment around bacterial cells^18^. In such conducive environments, crystal precipitation happens in the presence of any bivalent metal ions, such as Ca^2+^ or Mg^2+^, as shown in Fig. 1.

Despite existing studies on CaCO₃ polymorph (calcite, vaterite, and aragonite) selection^19–23^the mechanisms by which biological factors govern the formation of a specific polymorph or their combination are yet to be clearly understood. Remarkable biomineralised structures like nacre, seashells, and coral reefs feature an organised matrix of these polymorphs, interspersed with organic macromolecules, which control their formation and polymorph selection and enhance their multifunctionality^24–27^. Calcite is the most stable form of CaCO_3_ at Earth’s surface *P-T* conditions, while vaterite and aragonite are considered transient (metastable), forming under specific environmental conditions before converting to calcite^22,28,29^. Nature showcases fascinating examples of integrating various CaCO_3_ polymorphs with organic molecules. For instance, nacre, composed of approximately 95% aragonite and 5% organic macromolecules, has a brick-and-mortar structure that provides it with 3000 times higher fracture resistance than pure aragonite^30^. The nature of the CaCO_3_ polymorphs (stable vs. metastable, with equidimensional vs. fibrous morphology) might be a decisive factor in determining their performance and durability. Thus, comprehending how these polymorphs are selected in nature or whether this process can be mimicked using microbial systems is critical.

The major factors driving the morphology and phase selection of microbial CaCO_3_ biominerals^3,21,22,31–33^ are: (1) microbial metabolic pathways, (2) The mineralogy and surface physicochemical properties of the substrates, (3) The presence of organic macromolecules in the bacterial biofilms, such as extracellular polymeric substances (EPS) and other metabolites, and (4) The environmental conditions facilitating the precipitation, including pH, temperature (*T*), pressure (*P*), oxygen, moisture, and nutrients for the bacterial cells (illustrated in Fig. 1).

Some of these factors have been investigated in the literature. Interestingly, distinct microbes can produce different CaCO_3_ polymorphs under the same conditions^20,34^. Hammes et al. proposed that strain-specific mineralisation of CaCO_3_ precipitates, i.e., polymorph selection, is due to diversity in the urease expression coupled with the enzymatic activity^21^. The integral role of structural water and strain-specific amino acids in the formation of metastable vaterite has also been recorded^34^. Substrate mineralogy and its surface physicochemical characteristics are the other major factors that could influence polymorph selection in nature^22,35^. The studies on substrate mineralogy reveal that bacterial calcite grows more coherently over calcitic substrates due to a higher affinity for bacterial attachment than silicate substrates, where vaterite forms dominantly^22^. On the contrary, in a recent study, the majority of the precipitates were reported to be calcite irrespective of the mineralogy or surface characteristics of substrates, including silica sand, feldspar, kaolinite or montmorillonite, particularly in the presence of sea-water-based urea hydrolysis^35^. Therefore, it is critical to investigate the mechanism by which different microbes favour different polymorphs of CaCO_3_ during biomineralisation.

Moreover, the microbial biofilm significantly influences the regulation of crystal form and appearance^3,36–39^. Bacterial biofilms comprise microbial cells surrounded by a matrix of EPS^36,37,40,41^. Organic macromolecules (carbohydrates, proteins, nucleic acids and lipids/fats) are the key constituents of bacterial biofilms^36^. Biofilm is assumed to be essential for the extracellular activities of bacterial cells, such as the degradation of organic matter, nutrient exchange, and coordination of gene activities^36^. Moreover, biofilm provides the microenvironment to facilitate or inhibit the mineralisation process over micro-spatial scales^31,32,36,37^. EPS is the biopolymeric matrix secreted by microorganisms, comprising mainly carbohydrates (polysaccharides) and proteins^42^. EPS acts as an infrastructure holding the biofilm together. Apart from carbohydrates and proteins, EPS also contain lipids and organic acids (nucleic acids)^43^. Therefore, it is of critical interest to understand how EPS can influence the phase (i.e., polymorph) and morphology of the precipitates. Although previous studies have reported the critical influence of biofilm and EPS in bacterial mineralisation^3,38,44,45^their impact on CaCO_3_ polymorph selection via the ureolytic pathway has been limitedly explored.

This study advances current knowledge in bacterial biomineralisation processes by addressing the research gaps (as highlighted in blue text, factors 1 to 3 in Fig. 1), with a primary focus on the role of substrate mineralogy and organic macromolecules present within the EPS matrix in CaCO₃ polymorph selection at ambient pH and temperature employing two distinct microbes. The study employs an in-depth experimental investigation at micro to molecular scales using advanced tools such as Variable Pressure-Field Emission Scanning Electron Microscopy (VP-FESEM), zeta potential measurements, Raman spectroscopy, and Time-of-Flight Secondary Ion Mass Spectrometry (ToF-SIMS). To understand how the selection of bacterial CaCO₃ polymorphs takes place in nature, the specific objectives of the current study are defined as follows:

- To investigate the influence of different microbial metabolic pathways (ureolytic and EPS-assisted ureolytic),
- To examine the influence of substrate mineralogy and surface characteristics, and
- Tracking the adhesion of the organic macromolecules on the different geological substrates to determine their role in binding calcium ions.

This study highlights how advanced techniques such as Raman spectroscopy and ToF-SIMS can be crucially useful in tracking the role of organics in bacterial mineralisation processes.

## Materials and methods

### Bacteria characteristics

In this study, two ureolytic microbes were selected to distinctively highlight the influence of EPS on the precipitate’s morphology and mineralogy. The microbes were procured from the American Type Culture Collection (ATCC) for the current study. The conventional biocementing microbe *Sporosarcina pasteurii* (SP, ATCC 11859) was compared with the high EPS-producing microbe *Bacillus subtilis* (BS, ATCC 6633). The microbes were cultivated from a freeze-dried state to liquid nutrient broth media. The cultivated microbes were spread on the urea-agar base plate, and single colonies of the microbes were transferred for subculturing to nutrient broth-urea (NBU) media, where filter-sterilised 2 wt% urea was supplemented to the media post-autoclaving nutrient broth (NB) (13 g/l) in water. The analytical grade chemicals (NB, urea, urea-agar base, phosphate buffer and calcium chloride dihydrate) were procured from Sigma-Adrich, Australia. The microbes were sub-cultured three times before starting the experiment at 37 °C.

The growth characteristics of the bacteria were recorded with their optical density at 600 nm using a spectrophotometer (Thermo Fisher Scientific). The urease activity of the microbes was evaluated following the electrical conductivity method^46,47^ using a benchtop pH and conductivity meter (Thermo Fisher Scientific). Briefly, the phosphate-buffer saline solution (PBS)-washed cells were resuspended in fresh NBU media, and 2 ml of the bacterial culture was added to 18 ml of 1.1 M urea solution. Their electrical conductivity was measured for 10 min, and the urease activity was calculated considering the dilution factor^46^.

To quantify the biofilm and EPS, both the microbes (SP and BS) were cultivated for 48 h of bacterial growth in the NBU media. Biofilm formation was quantified with the crystal violet staining assay^48,49^. Each bacterial strain, diluted to an OD₆₀₀ of 0.5 in the NBU media, was inoculated into microtiter plates (1 µL culture in 99 µL medium) and incubated at 37 °C for 48 h. Plates were sealed with parafilm, and after incubation, wells were rinsed thrice with sterile water to remove unattached cells and then air-dried. Biofilms were stained with 0.1% crystal violet for 45 min, the excess dye was removed by rinsing, and the stain was solubilised in 95% ethanol at 4 °C for 30 min. Finally, 100 µL from each well was transferred to a new plate, and the optical density was measured at 570 nm (OD_570_) using a microplate reader. The EPS was extracted following the chilled ethanol method^33^. 100-ml bacterial culture after 48 h of growth was centrifuged at 13,200 g for 25 min at 4 °C, and the supernatant was stored at − 20 °C. EPS was precipitated from 50-ml supernatants by adding three volumes of chilled ethanol, incubating at 4 °C overnight, before centrifuging. The centrifuged pellets were dried at room temperature for 24 h and weighed.

### Substrate characterisation- X-ray diffraction and zeta potential

Three natural geological mineral substrates (apatite, A; calcite, C; and quartz, Q) were selected to investigate the influence of their mineralogy and surface chemistry on the bacterially induced precipitates’ morphology and mineralogy. Calcite, apatite, and quartz were selected as they represent distinct mineral classes (carbonates, phosphates, silicates) and are commonly found in geological formations such as calcareous soils, silica sand and phosphate rocks. The substrates (crystals of size 1 cm to 3 cm) were procured from Amy’s Crystals, Australia. The aggregates were powdered into a grain size < 2 μm with a ring mill for their characterisation. The mineralogical composition of the substrates was evaluated using quantitative X-ray diffraction (XRD) on a Bruker advanced diffractometer equipped with Nickel-filtered Cu-Kα radiations (λ = 1.5405 Å), covering a 2θ range of 5° to 90° with a step size of 0.013°. Their zeta potential was evaluated on a Malvern Zetasizer Nano-Zs with an aliquot prepared by suspending the powdered substrates in ultrapure water. The measurements were taken in triplicate after 24 h of aliquot preparation, and their pH was recorded using an Orion pH electrode.

### Sample preparation

The microbial cells in the logarithmic phase (O.D._600_ ≈ 0.5) were washed with PBS (0.01 M) and centrifuged before being suspended in fresh NBU media for application to the substrates. Sterile conditions were maintained throughout the experiments to avoid the influence of undesired contamination on EPS formation or biomineral precipitation. The substrates were stored in sterile Petri dishes sealed with parafilm to prevent the evaporation of bacterial or cementation droplets throughout the experiment.

To analyse the influence of microbial pathways on precipitates’ morphology, 50 µL of bacterial solution (SP and BS) was dropped on coverslips. A 24-h bacterial retention period was allowed to enable interaction between the bacteria and the substrates and to facilitate biofilm formation. After 24 h, 250 µL of mineralisation media (2 wt% nutrient broth, 500 mM urea and 500 mM CaCl_2_) was dropped on the coverslips. 2% nutrient broth was supplemented in the cementation media to support the bacterial cells for their metabolic activity. A 500 mM cementation media solution (CaCl_2_ and urea) was considered based on the previous studies on engineering applications with the ureolytic pathway^20,50^. The samples were kept in sterile Petri dishes at 37 °C for a total of 48 h (24 h of bacterial retention and 24 h of mineralisation) for the evaluation of their metabolic activity and biomineral precipitation. The Petri dishes were sealed with parafilm to minimise evaporation. Multiple precipitation cycles (4 cycles) were also conducted on the coverslip by drop casting the mineralisation media, maintaining a 24 h retention interval for each cycle.

To study the influence of the mineral substrates on the morphologies of the precipitates, the substrates (in aggregate form) were cut, polished and mounted in epoxy resin (30 mm diameter, 6 mm depth). The specimens were sterilised with 70% ethanol and washed with autoclaved ultrapure water. The bacterial solution (50 µL) was dropped on the substrates (A, C and Q). To facilitate microbial fixation on the substrate surface, the specimens were incubated at 37 °C for 24 h in sterile Petri dishes sealed with Parafilm to minimise evaporation. After 24 h, 250 µL of mineralisation media (2 wt% nutrient broth, 500 mM urea and 500 mM CaCl_2_) was dropped on the substrates. The substrates with low-EPS-producing bacteria (SP-A, SP-C, SP-Q) and abundant EPS-producing bacteria (BS-A, BS-C and BS-Q) were kept in sterile Petri dishes at 37 °C for 48 h for the evaluation of their metabolic activity and biomineral precipitation.

All samples were gently rinsed with ultrapure autoclaved water after the precipitation period and air dried in sterile conditions (biosafety cabinet) before mineralogical and microstructural analysis.

### Test procedure details

#### Morphological investigation with VP-FESEM

One of the concerns in the current study with using conventional high-resolution scanning electron microscopy was the need to dry the specimens before coating them with a conductive layer (such as platinum, gold, or carbon) to prevent charging, which could result in the loss of information on the biopolymeric EPS matrix. To tackle the challenge, the air-dried specimens were studied under high-resolution VP-FESEM Tescan Mira3 XMU without any coating. Energy Dispersive X-ray spectroscopy (EDS) was also performed at selected crystal points to confirm the precipitates’ elemental composition.

#### Mineralogical investigation through raman spectroscopy

Raman spectroscopy offers an advantage over XRD for biological specimens as it is a quick analytical technique that can analyse the specimen without altering or drying and yields molecular information from amorphous and crystalline organic and inorganic materials. This is particularly useful for investigating polymer-mineral interactions. The Raman spectroscopy in the current study was performed using a WITec alpha 300SAR equipped with an NdYAG laser of wavelength 532 nm (green) and of 50 mW power. Silicon was used as a reference material for calibration of the spectrometer. Single-point spectra were obtained employing 100 accumulations at 0.1 s integration time.

#### Molecular tracking of organics (biofilm) entrapped on substrates through ToF-SIMS

The precipitated biominerals were analysed employing a surface-sensitive analytical technique with an IONOTOF M6 ToF-SIMS instrument equipped with a 30 kV Bi ion gun for high-resolution spectrometry and imaging to investigate the adhesion of organic macromolecules on different substrates and check their ability to immobilise calcium ions. The instrument uses a high current ion for surface cleaning with two ion sources: oxygen (O_2_^+^) and cesium (Cs^+^) ion source under ultra-high vacuum. Data were collected from m/z = 0–200 for positive and negative ions. Positive SIMS spectra were calibrated with CH_3_^+^, C_2_H_3_^+^, Ca^+^, and CaOH^+^ peaks, while negative spectra used CH^−^, O^−^, OH^−^, and CO_3_^−^ peaks. Post-calibration mass differences were below 20 ppm.

The pressure in the vacuum chamber was maintained at 10^−9^ mbar during the analysis to reduce the influence of any residual gas. The sample surface was subjected to a primary ion beam, which produced secondary ions (SI), as well as neutral atoms, electrons, photons, and both atomic and molecular charged ions. Only the charged secondary ions were detected by the ToF (time-of-flight) detector. After travelling through the flight tube, these charged ions were separated based on their mass-to-charge ratio (m/z). The results were analysed using the SurfaceLab 7.2 software.

It is to be noted that ToF-SIMS is a powerful tool that has the capability of high-resolution tracking of organic molecules, which, unlike EDS, is not restricted to elemental mapping. Resin-mounted substrates with a single mineralisation cycle were carefully attached to the sample holder using clean copper conductive tape without any pretreatment. The selection of a run with just one mineralisation cycle strived to disclose the EPS-Ca mineral interaction at the very early stages of bacterial biomineralisation of CaCO_3_ on the apatite and calcite substrate. Only the high-EPS producing BS strain was examined with ToF-SIMS, as SP produces negligible EPS comparatively. The quartz substrate was not analysed with ToF-SIMS due to the fact that no significant changes in bacterial calcium carbonate precipitates were observed when compared with the apatite mineral substrate.

#### EPS characterisation- total carbohydrates, total protein and functional groups through colourimetry protocols and fourier transform-infrared spectroscopy (FTIR)

 The dried EPS extracted using the chilled ethanol method^33^as outlined in Sect. 2.1, was evaluated for its total carbohydrate and protein content using established biochemical methods. Total carbohydrate content was determined using the phenol-sulfuric acid assay, following the protocol described by Dische et al.^51,52^. In this assay, 50 µL of EPS was mixed with 50 µL of 5% phenol and 500 µL of concentrated sulfuric acid, incubated at room temperature for 30 min, and the absorbance was measured at 490 nm. A glucose standard curve (0–100 µg/mL) was used for quantification. Protein content was measured using the Lowry method^53^with bovine serum albumin (BSA) serving as the standard for calibration. For each assay, 50 µL of the sample was mixed with alkaline copper reagent, incubated for 10 min at room temperature, then reacted with Folin–Ciocalteu reagent, and absorbance was measured at 750 nm. A BSA standard curve (0–100 µg/mL) was used for quantification.

To understand how the functional groups comprising microbial-EPS influence the phase selection and morphology of the precipitated mineral, the dry powdered EPS was analysed on a Bruker IFS66 FTIR spectrometer with a wavenumber range of 400 to 4000 cm^−1^ and 20 consecutive scans at a resolution of 4.0 cm^−1^ in absorbance mode.

## Results

### Bacterial growth characteristics, urease activity and EPS production rate

**Fig. 2:**
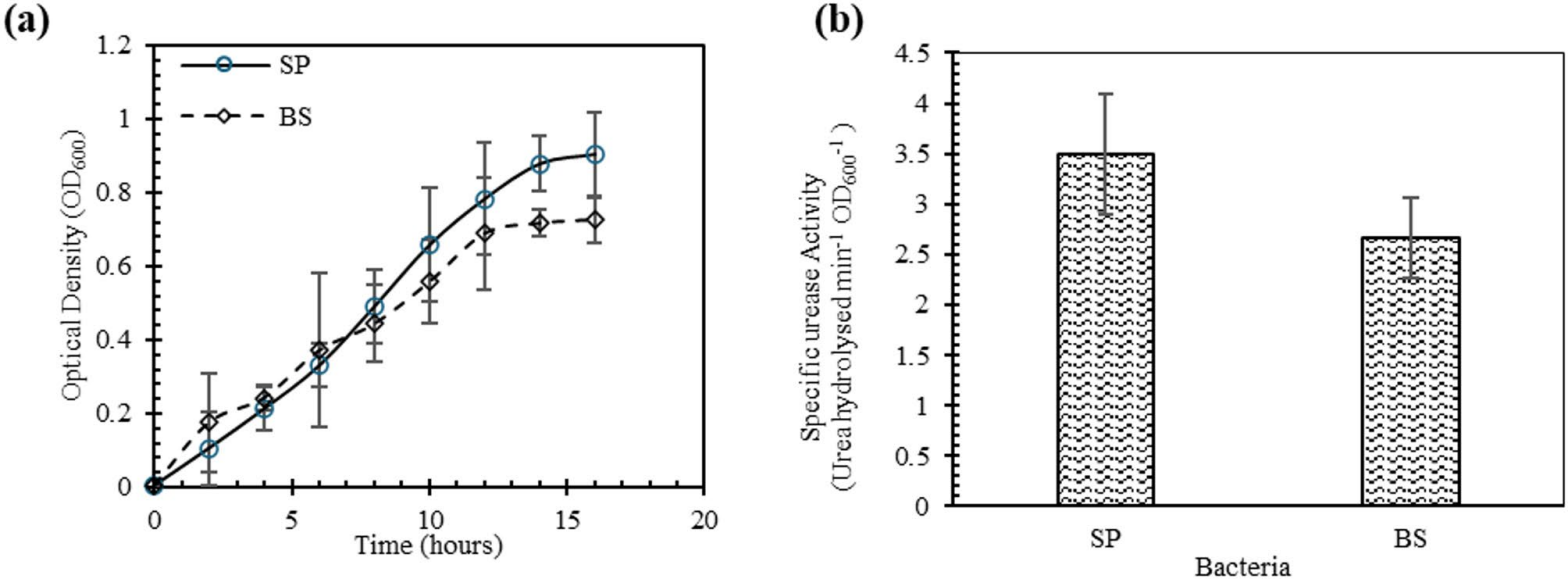
(**a**) Growth characteristics; and (**b**) Specific urease activity of SP and BS. Error bars show standard deviation (1σ).

The growth curve of the bacteria in the NBU media is reported in Fig. 2 (a). Within 18 h of subculturing, the microbes gained an optical density (OD_600_) of ~ 1. The cultures in the logarithmic phase were centrifuged, washed with phosphate buffer saline solution (0.01 M) and resuspended in fresh NBU culture for their experimental application. The specific urease activity of the standard strain SP was 30% higher than BS, as demonstrated in Fig. 2 (b). However, both strains were potent for bacterial mineralisation as their urease activity was greater than 2.5 mM urea hydrolysed per min for unit OD_600_. The urease activities of ureolytic strains have been reported to vary from 1.78 to 9.7 mM urea hydrolysed min^−1^ O.D._600_^−1^ for the engineering applications in the previous studies^46,47,54^. Therefore, both microbes are capable of degrading urea to facilitate calcium carbonate mineralisation.

**Fig. 3:**
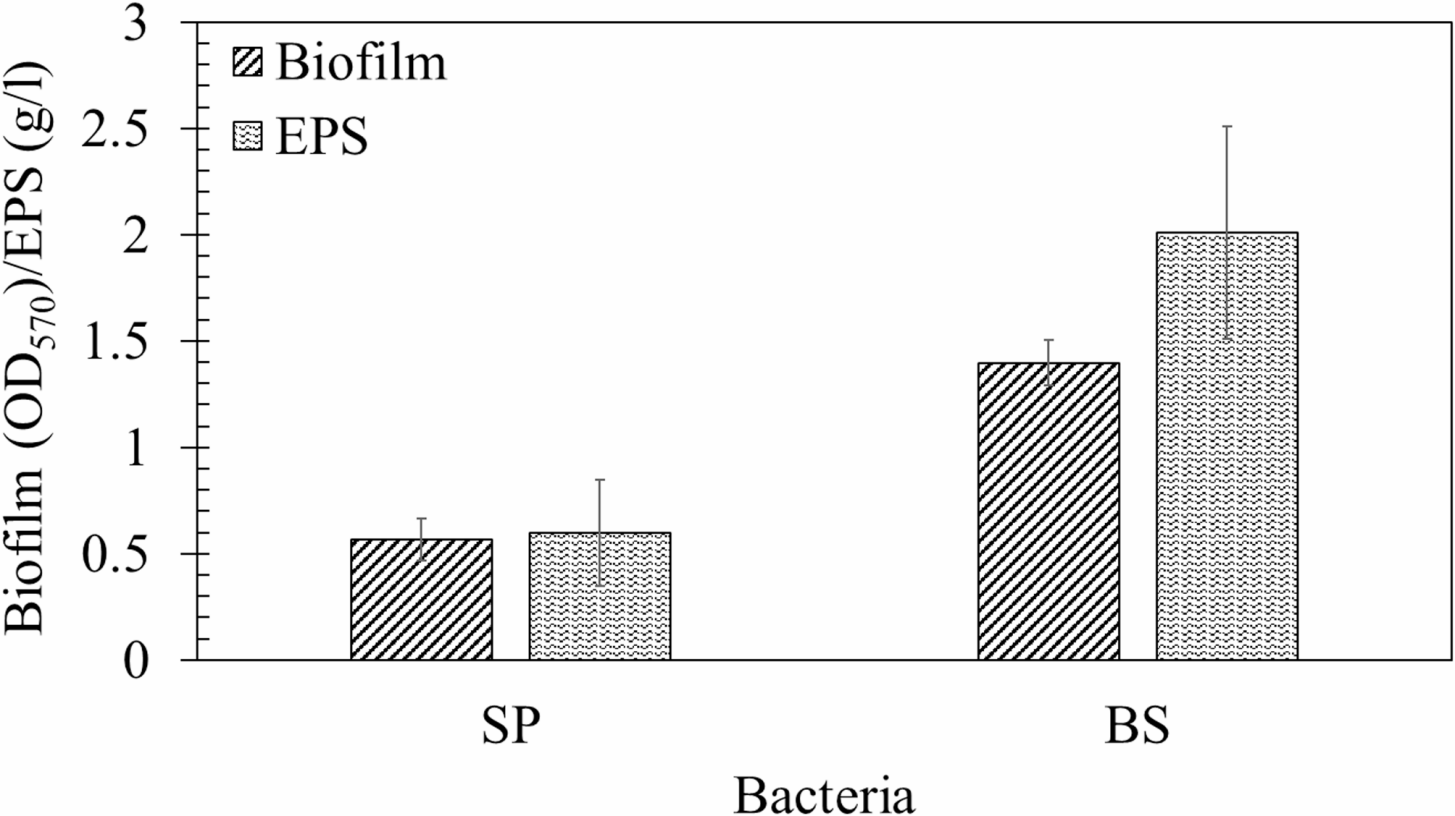
Biofilm (in OD_570_) and EPS (in g/l) production capacity of the selected microbes. Error bars show standard deviation (1σ).

SP has been used for biomineralisation in numerous studies^55–57^. Figure 3 illustrates the biofilm produced by SP and BS. The amount of biofilm produced in SP (OD_570_ = 0.56) is determined to be significantly lower in comparison to BS (OD_570_ = 1.4). There are a few studies on the role of biofilms in the biomineralisation process of SP, suggesting biofilms act as nucleation points^31,37,58^. Similar trends were observed in the EPS quantity produced by the microbes. Prior studies based on the conventional ureolytic microbe *Sporosarcina pasteurii* have limitedly discussed the role of EPS in the biomineralisation process, most likely because of negligible EPS production by this bacterium in comparison to BS. Indeed, SP produces almost negligible EPS as compared to BS, as shown in Fig. 3. In prior studies on *Bacillus* strains, the EPS produced ranged from 1 g/L to 8 g/L^33^^,^^59^.

### Substrate characterisation

The natural substrates selected in the current study were observed to be apatite (A), calcite (C), and quartz (Q). The substrates’ photographs and X-ray diffraction patterns are shown in Supplementary Fig. S1 and Fig. S2. Zeta potential is a measure of the electrostatic potential at the slipping plane near the substrate’s surface. The average zeta potential values of the A, C, and Q substrates were − 21.2 mV, −0.364 mV and − 21.5 mV. The average pH values of the aqueous dispersion aliquots of A, C and Q (after 24 h of aliquot preparation) were 7.5, 8.1 and 7.1, respectively. The notable rise of pH in the case of calcite is most likely due to the dissolution of carbonate ions and structural defects in the crystal lattice due to random natural anomalies or substitutions^60^. Defects in the CaCO₃ crystal lattice can lead to residual surface charges and promote ion exchange with the solution, influencing surface reactions such as hydrolysis or protonation, which in turn can alter the local pH^60^. These surface heterogeneities enhance reactivity, making the surrounding solution more sensitive to pH shifts.

### Influence of microbial pathways on CaCO_3_ polymorph selection

**Fig. 4:**
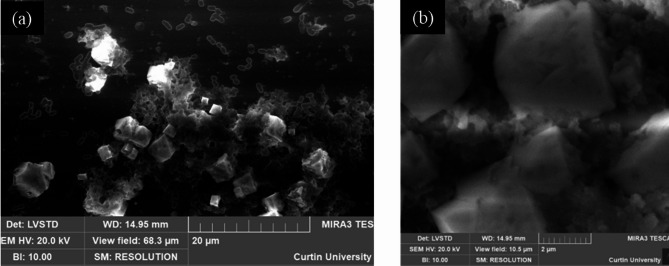
Rhombohedral CaCO_3_ (calcite) precipitates formed by SP (*Sporosarcina pasteurii*) (**a**) low magnification image showing precipitates and bacterial debris; (**b**) High magnification image showing rhombohedral crystals. The images were taken under variable pressure and backscattered mode to minimise charging; however, it was implausible to avoid it in most cases.

To evaluate the influence of microbes (abundant vs. low EPS) on the CaCO_3_ polymorph selection, preliminary investigations were conducted on the glass coverslips (a control substrate). Figure 4 (a) and (b) show representative crystals precipitated with low-EPS-producing SP. They are rhombohedra, in some cases with truncated or rounded faces, likely due to interaction (adsorption) with organic by-products of bacterial activity. Such euhedral and subhedral crystals are embedded in a matrix of bacterial cells and cell debris. Based on this distinctive rhombohedral habit, it is deduced that the polymorph is calcite, which is in agreement with the literature^19,20,61^. The polymorph of the CaCO_3_ precipitate was also confirmed with Raman Spectroscopy (Supplementary Fig. S3 a). The spectrum exhibited a main v_1_ band at 1089 cm^−1^, a small v_4_ band at 715 cm^−1,^ and the lattice vibration bands at 156 and 262 cm^−1^, all of them being distinct signatures of calcite^62^. The size of crystals varied from 2 μm to 10 μm.

**Fig. 5:**
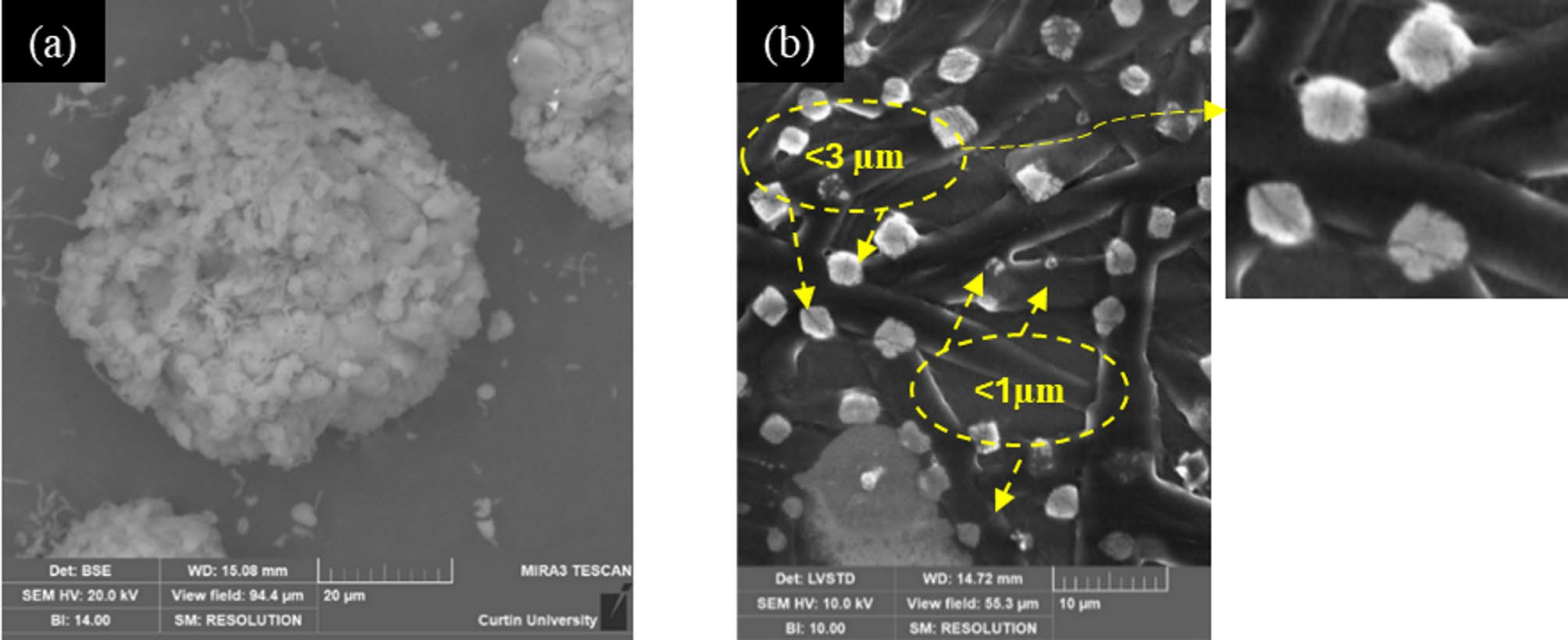
(**a**) Spheroid vaterite precipitates by BS (*Bacillus subtilis*) and (**b**) Mesocrystals anchored in the EPS matrix.

In contrast, BS predominantly precipitated large-sized spheroidal (20 μm to 100 μm) crystals on the coverslips, as shown in Fig. 5 (a). EDS analysis performed on a random point of the precipitates revealed their elemental composition to be rich in calcium, carbon and oxygen, indicating the crystals to be calcium carbonate. The spheroidal shape and rough texture suggest vaterite precipitation^38^. The mineralogy of these precipitates was confirmed with Raman spectrometry, showing the distinctive bands of vaterite: the v_4_ doublet at 739 and 749 cm^−1^ and the intense v_1_ doublet at 1075 and 1090 cm^−1^ (supplementary Fig. S3 b)^63^. Around the periphery of the spheroidal vaterite precipitates shown in Fig. 5 (a), a network anchoring sub-micron CaCO_3_ mesocrystals was observed (Fig. 5b). These mesocrystals (< 3 μm) are suspected to be associated with an extracellular polymeric substance (EPS)-like matrix; however, due to their small size and limited observations, detailed morphological characterisation was not plausible.

**Fig. 6:**
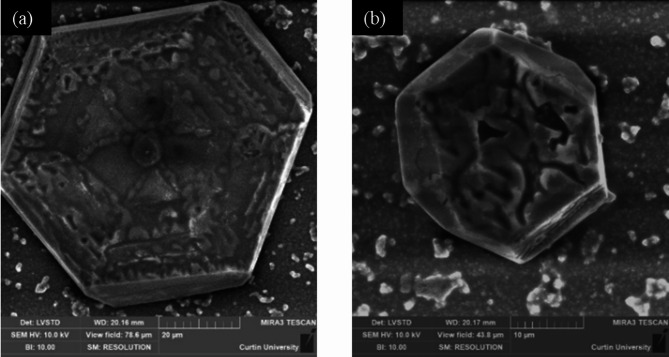
Pseudo-hexagonal vaterite structures (**a** and **b**) observed with BS.

Mesocrystals are characterised by ordered vectorially aligned anisotropic submicron to nanoscale particles held together by surface-bound polymeric fibres^64–66^which are evident in Fig. 5b. It is to be noted that the mesocrystals deposited on the polymeric fibres are the individual crystals in Fig. 5b, which include the organics among nanodomains (darker areas in the magnified inset). The large micrometre-sized fibres among individual mesocrystals do not contribute to the formation of mesocrystals. They are most likely the rest of the EPS to which the mesocrystals are attached. Biofilm and EPS characterisation revealed that the BS system produces substantially more EPS than SP. This abundant organic matrix likely plays a key role in stabilising vaterite and facilitating its self-assembly as a mesocrystal structure^38^. This observation is consistent with the findings of Rodriguez-Navarro et al.^38^ and Xu et al.^65^. Euhedral pseudo-hexagonal-plate-like precipitates (identified as vaterite) were also observed to form in the presence of abundant EPS produced by BS (Fig. 6a, b).

**Fig. 7:**
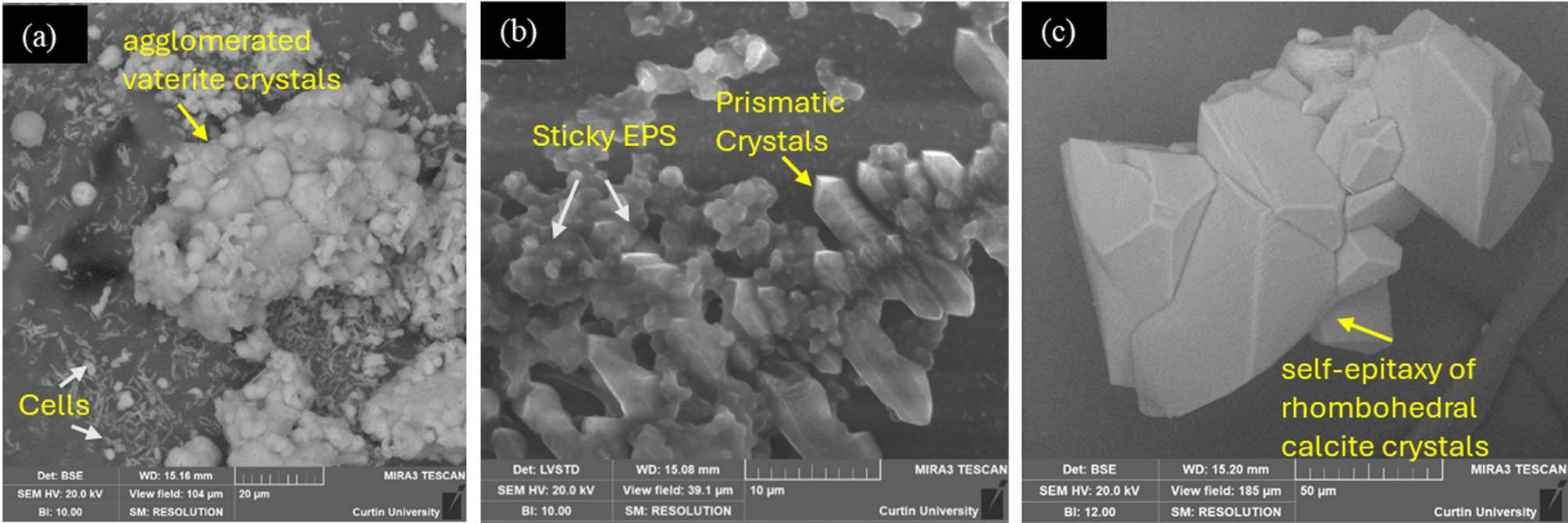
Bacterial calcium carbonate biomineralisation following multiple cycles. (**a**) BS-generated agglomerated vaterite crystals; (**b**) BS-generated EPS coupling CaCO_3_ crystals grow as dendrites on a coverslip substrate; and (**c**) Aggregated CaCO_3_ (calcite) crystals formed in the presence of SP.

Further microscopic analysis of the biominerals precipitated on coverslips with SP and BS after 4 mineralisation cycles revealed agglomerated vaterite crystals (Fig. 7a) and elongated prism-shaped crystals associated with EPS (Fig. 7b) in the case of BS, whereas in the case of SP, the self-epitaxy of CaCO_3_ successively growing over the mineralisation cycles was evident (Fig. 7c). Elongated prism-shaped crystals are possibly generated with the EPS because the negatively charged functional groups in EPS guide oriented crystal growth via electrostatic interactions, promoting anisotropic morphologies. As shown in Fig. 7b, the microscopic analysis highlights the sticky EPS matrix anchoring the precipitates in different orientations. The structures in Fig. 7b show a 2D dendritic (fractal) shape, like that of snowflakes, oxides or salt crystals formed on a substrate at very high supersaturation following rapid evaporation/cooling, nucleation, self-assembly and fast crystal growth^67–69^. These features suggest that the biofilm and EPS produced by the BS cells can induce a very high supersaturation during their metabolic activity, specifically with the multiple treatment cycles (i.e., they display high metabolic rates). Previous studies also reported that biofilms (EPS) create chemical gradients^32,70^ along with localised supersaturation conditions, which is most likely the reason for the formation of the irregular or fractal-shaped crystals shown in Fig. 7 (b). In this precipitation process, EPS appears to play a crucial role as a coupling agent between the substrate and the bacterial calcium carbonate. In contrast, SP, despite its higher ureolytic activity and greater carbonate availability, facilitates a uniform supersaturation, leading to nucleation and layer-by-layer growth of CaCO₃, in agreement with previous literature^71,72^as shown in Fig. 7 (c).

### Influence of substrate mineralogy on biomineral phase and morphology

**Fig. 8:**
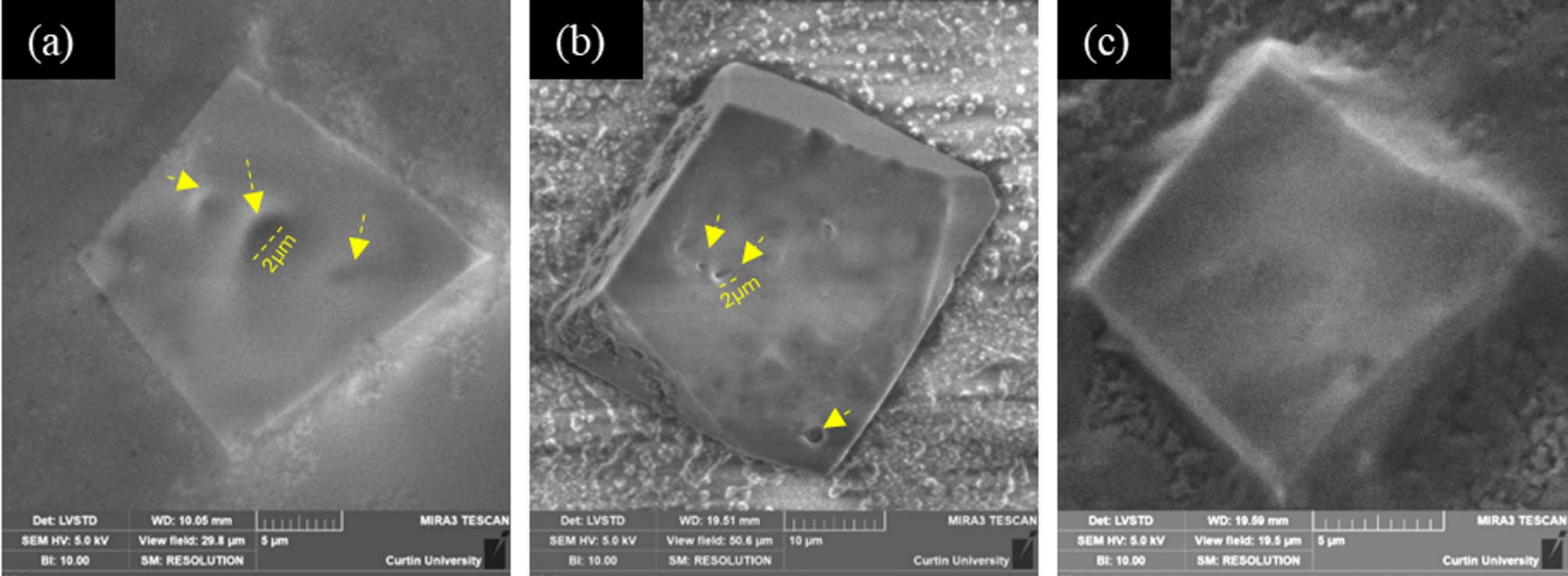
Biomineralisation of calcite with SP on (**a**) apatite, (**b**) calcite, and (**c**). quartz (the yellow arrows show imprints suggesting possible bacterial entombment).

After evaluating the influence of bacterial pathways (low vs. abundant EPS production) on calcium carbonate precipitation over glass coverslip (a control substrate), the influence of the mineralogy and surface properties of three distinct natural substrates (apatite, calcite and quartz) was investigated. In the case of SP, no significant difference was observed in the mineralogy of the precipitates, which predominantly consisted of calcite with mostly rhombohedral morphology, as shown in Fig. 8 (a-c). However, the calcite crystals formed on the calcite surface exhibit facets, as shown in Fig. 8b, possibly due to the difference in zeta potential magnitude of the different substrates. It is to be noted that low-magnification images could not be captured due to inadequate clarity due to the low vacuum in the VP-FESEM setup at the time of image capture. The size of the crystals varied in a narrow range from 20 μm to 40 μm. The precipitated minerals were pitted by bacteria-shaped holes (bacterial imprints) that have an approximate size of bacterial cells (as shown in Fig. 8a- b), suggesting that the bacteria likely got entombed while precipitating the crystal around them before eventually getting lysed. The Raman spectra of these precipitates resulted in identical spectra to those in Fig. S3a, confirming their calcite mineralogy. In contrast, XRD analysis did not show Bragg peaks of calcite in these samples due to a very small amount of precipitates.

**Fig. 9:**
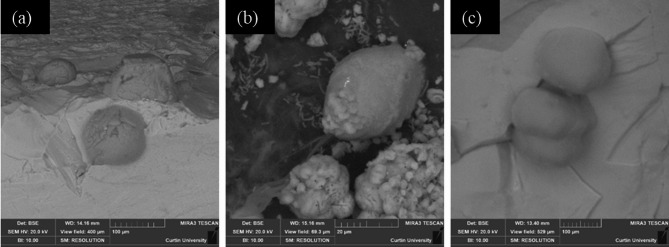
Biomineralisation of vaterite spherulites/spheroids with EPS producing BS on (**a**) apatite, (**b**) calcite, and (**c**) quartz.

In the case of BS, the precipitates were found to be spheroids (50–100 μm), as illustrated in Fig. 9 (a-c), in contrast to the rhombohedral precipitates produced by SP. Moreover, elongated/lens-shaped vaterite was observed on the calcitic substrate along with bacterial debris, as shown in Fig. 9b, in comparison to the apatite and quartz substrates. The spheroidal shapes of the precipitates suggested them to be vaterite^20,38,73^. The mineralogy of the precipitates was confirmed with Raman analysis, resulting in a similar vaterite spectrum as in Fig. S3b. It is to be noted that the biomineralised precipitates on the substrates were washed with deionised water and dried prior to Raman analysis to conveniently locate the precipitates and avoid complex, unidentifiable spectra on the natural substrates due to the metabolites and EPS.

Altogether, these results underline that the dominating factor for polymorph selection (also affecting crystal shape) is the metabolic features of the two bacterial strains. In particular, the capacity of BS to generate abundant biofilm and EPS appears to be the overruling factor determining the formation of vaterite structures, as opposed to SP, which generates negligible amounts of biofilm and EPS, thereby favouring the formation of calcite.

### Tracking biofilm, EPS and bacterial carbonates on the substrates with ToF-SIMS

**Fig. 10:**
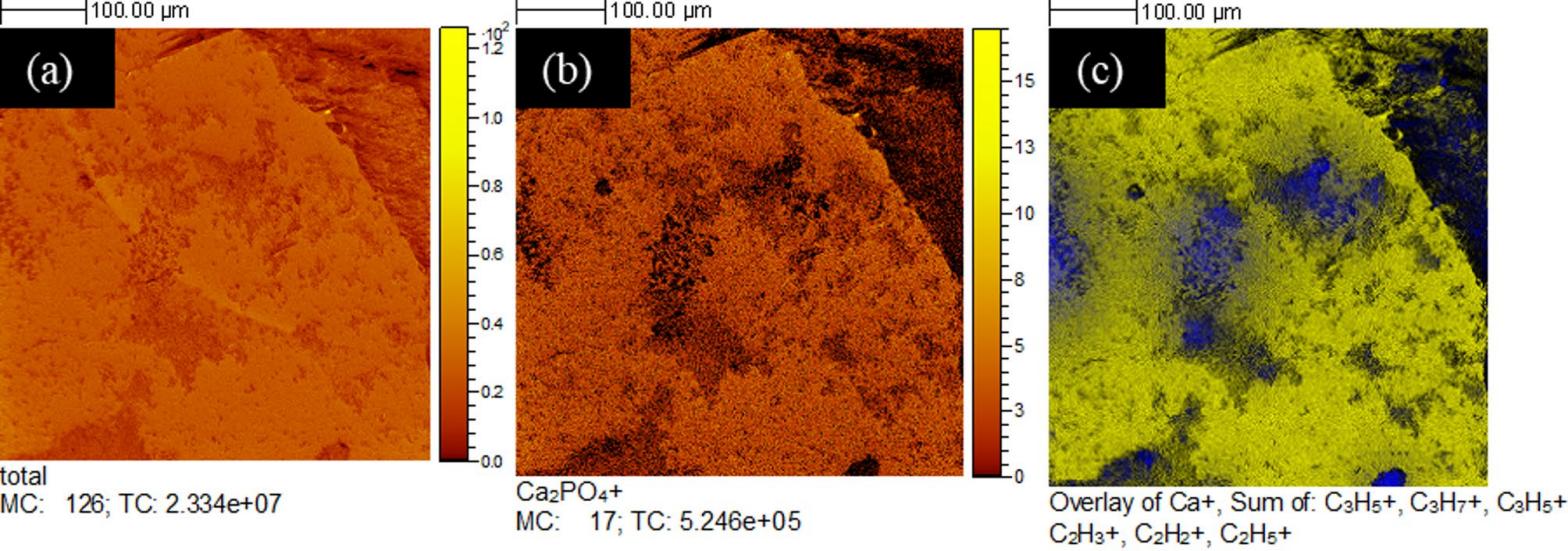
ToF-SIMS elemental maps. (**a**) Total ion concentration; (**b**) Apatite substrate (Ca_2_PO_4_^+^) concentration; and (**c**) Overlay image showing the spatial distribution of calcium ions (Ca²⁺) and organic matter (carbohydrate-rich matrix). Blue represents the organic matrix, while yellow indicates regions with total calcium ions. The green interface is possibly due to the overlap of calcium ions adsorbed onto the organic matrix (yellow + blue).

A ToF-SIMS analysis was performed to characterise the structural features of the organic (biofilm and EPS) matrix and its relationship to Ca-mineralisation. The organic matrix was examined by mapping the organic molecules (carbohydrates) around the bacterial CaCO_3_ precipitates. Since biofilm loses substantial moisture during the drying process in an ultra-high vacuum, the information regarding the EPS network structure could not be properly examined through ToF-SIMS. An overlay of organic compounds along with Ca^2+^ ions was constructed. Figure 10 shows the maps corresponding to the run with the apatite substrate. Figure 10a presents the total concentration of ions on the substrate. The distinct Ca_2_PO_4_^+^ ions in apatite are illustrated in Fig. 10b. The overlay of Ca^2+^ and organics (different carbohydrate groups) is shown in Fig. 10c. The yellow tint in Fig. 10c shows the distribution of calcium ions and the organic matrix is marked with a blue region. The greenish tint in Fig. 10c shows an overlap of yellow and blue colours, suggesting that calcium ions are adsorbed on the organic matrix. The specific co-localisation of Ca signals (greenish tint) with regions enriched in organic material (along with the morphology and mineralogy confirmation by complementary Raman analysis shown in Fig. S3 and VP-FESEM) supports the interpretation that a significant portion of this calcium is adsorbed onto the organic matrix. Furthermore, given that ToF-SIMS is a surface-sensitive technique probing only a few nanometres into the sample and that the EPS or biofilm layer is considerably thicker (hundreds of nanometres to micrometres), the detected Ca signal must originate from the outermost organic layer, i.e., the EPS interface. This strongly suggests binding or association of Ca within the EPS matrix rather than from a bulk mineral phase alone.

In this case, the carbohydrate groups are fingerprints of the biofilm and EPS over the calcium ions. It is important to note that in Fig. 10(a), (b), and (c), with the same field of view, the visual differences result from the different ion distributions. The presence of Ca₂PO₄⁺ ions characteristic of the apatite substrate is shown in Fig. 10 (b) to aid in differentiating substrate-derived calcium from biomineralised calcium.

**Fig. 11:**
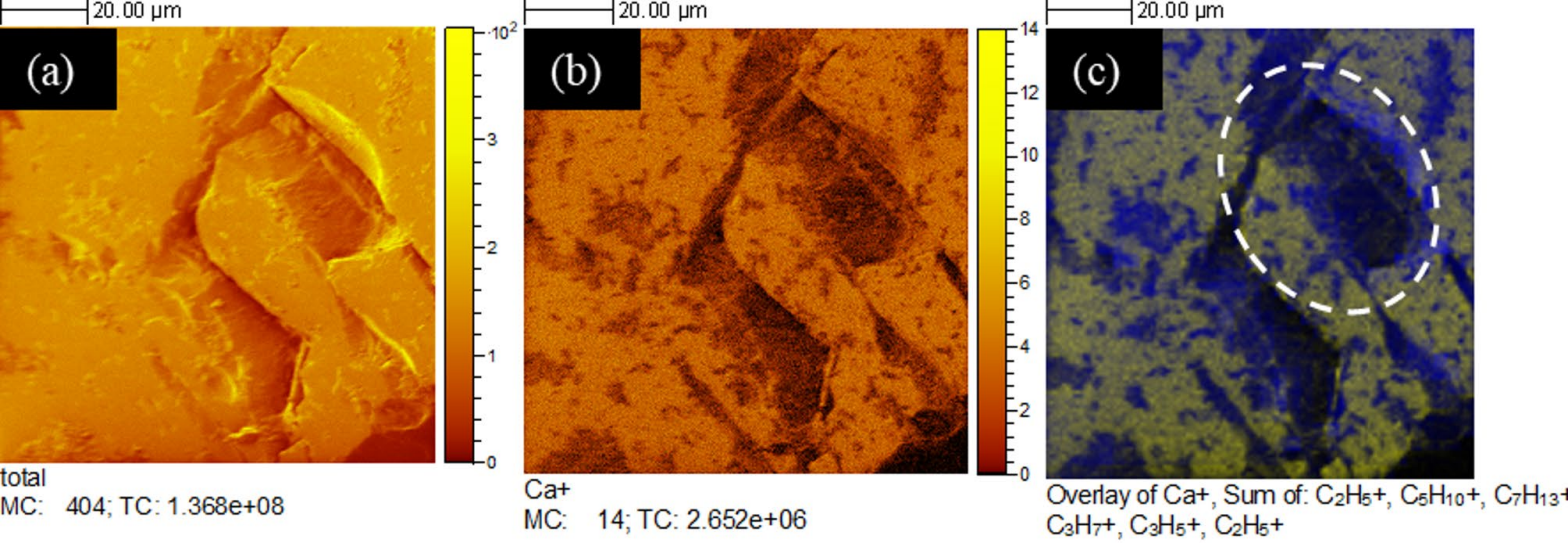
ToF-SIMS elemental maps. (**a**) Total ion concentration; (**b**) Calcite substrate (Ca^2+^) concentration and (**c**) Overlay image showing the spatial distribution of calcium ions (Ca²⁺) anchoring on organic matter (carbohydrate-rich matrix). Blue represents the organic matrix, while yellow represents calcium ions.

The organic matter attached to the calcite substrate is illustrated in Fig. 11. The total ion and Ca^2+^ ion concentrations (in yellow) are mapped in Fig. 11a and b, respectively. Figure 11c shows an organic matrix (in blue colour) anchored over the calcium ions (yellow). Adsorption of calcium ions was not clearly observed in the organic matrix, as shown in Fig. 11c, possibly due to strong Ca^2+^ ion interferences from the calcitic substrate. MC values stand for maximum counts in a pixel, whereas TC stands for total counts in the whole image in Figs. 10 and 11. The values are useful parameters to define the quality of an ion image produced.

It is important to note that while ToF-SIMS offers high surface sensitivity (limited to just a few nanometres), it does not have the capability to directly determine the mineral phases such as calcite or vaterite. Instead, it provides information on the distribution and intensity of secondary ions ejected from the surface upon sputtering. Therefore, information on mineral phases (calcite/vaterite) could not be determined, and the confirmation relies on the complementary VP-FESEM and Raman spectroscopy. Additionally, there were two challenges in adopting this approach: first, to precisely locate the small number of biominerals over the substrates within the ToF-SIMS setup, which has lower microscopic visualisation capabilities compared to FESEM, and second, the strong calcium signal interference, as the substrate was also calcium-rich.

In future studies, these challenges could be addressed by using FIB-SEM (Focused Ion Beam–Scanning Electron Microscopy) to mark the biomineralised regions prior to analysis and by incorporating a calcium isotope tracer (e.g., Ca-44) in the microbial cementation medium to aid in distinguishing substrate-derived signals during ToF-SIMS analysis.

### EPS composition and EPS-calcium carbonate biominerals interaction

**Fig. 12:**
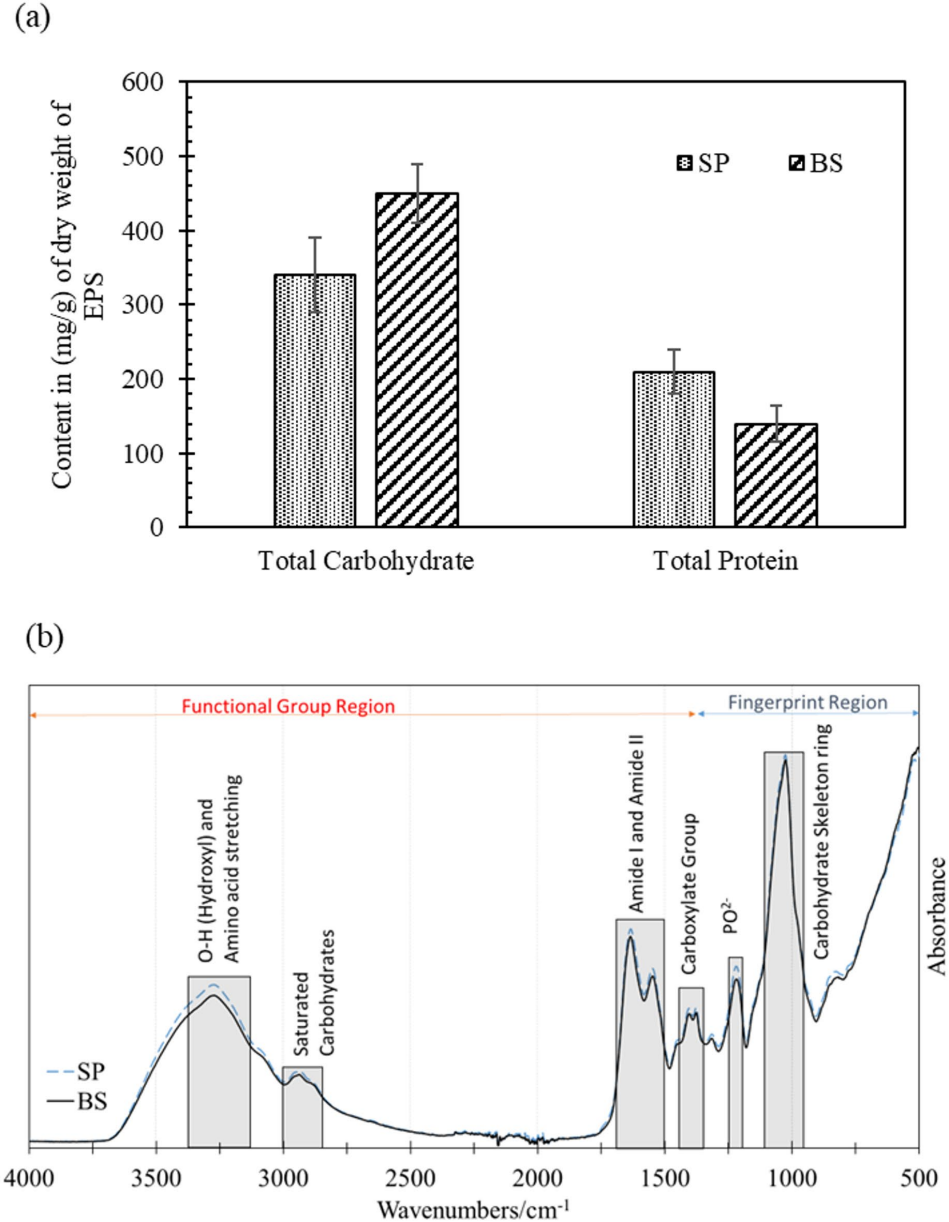
Analysis of EPS. (**a**) Total carbohydrate and protein content in the dry pellet of EPS produced by SP and BS; and (**b**) FTIR spectra of EPS produced by BS and SP. The functional groups of EPS are indicated by the shaded areas.

EPS was extracted from the BS and SP in the NBU media after 48 h of cultivation time and air-dried prior to evaluation of the total carbohydrate and total protein content, illustrated in Fig. 12a. It is to be noted that the quantity of extracted EPS in SP was significantly low (0.6 g/l) when compared with BS (2.01 g/l), as shown in Fig. 3. Moreover, the carbohydrate and protein content of the EPS from these microbes exhibited notable dissimilarities, as shown in Fig. 12a. EPS produced by BS has a notably higher (32% higher) carbohydrate content than SP, whereas the total protein content was 50% higher in the EPS produced by SP than BS. To further investigate the functional groups liable for the CaCO_3_ polymorph selection, the EPS produced by SP and BS were analysed with FTIR (Fig. 12b). The FTIR spectra of extracted EPS from BS and SP are almost identical. Although it is evident that the microbes influence the quantity of EPS produced, as well as the total carbohydrate and total protein content, the FTIR spectra reveal that EPS produced from both microbes have similar functional groups. The shaded areas in Fig. 12b highlight the relevant absorbance bands of the functional groups. The absorption bands in the region 3300–3500 cm^−1^ are assigned to hydroxyl (O-H) and amino groups (N-H) vibrational stretching^74^. The O-H stretching might be caused by the adsorbed water. The distinct small bands between 2890 and 2950 cm^−1^ corresponded to the C-H stretching of saturated carbohydrates^75^. The bands around 1650–1550 cm^−1^ corresponded to amide groups. The amide group is associated with proteins in the EPS matrix. The bending vibrations of the carboxylate group (C = O) were observed at around 1430 cm^−1^. The absorbance band around 1250 cm^−1^ is due to asymmetric stretching vibrations of PO^2−^ molecules^75^. The PO^2^⁻ bands, attributed to the external bacterial cell layer, muramic acid in peptidoglycan, and lipopolysaccharides, showed significantly higher intensities in the EPS produced by mineral-forming strains compared to those from the non-mineral-forming strain under tested conditions^75,76^. The highest absorbance band with peaks ranging from 1060 to 1070 cm^−1^ is attributed to the complex vibrations of the carbohydrates with skeleton ring structure^77^.

## Discussion

This study explored the process of bacterial mineralisation of calcium carbonates via two bacterial routes, with little and abundant EPS production, respectively, on three different substrates, namely apatite, calcite, and quartz. The bacteria chosen had distinct EPS production capacities and enzyme activity, both in the NBU media. The substrates present distinct zeta potentials at neutral to slightly alkaline pH, indicating that their surfaces possess different surface charge distributions and exhibit varied electrostatic interaction capabilities. The bacterial mineralisation process was investigated through single and multiple biomineralisation cycles. Extensive microscopic and mineralogical analyses were performed using cutting-edge techniques, including VP-FESEM, zeta potential measurements, ToF-SIMS, Raman spectroscopy and FTIR. The major findings from the current study and their implications are discussed below.

### Influence of substrate characteristics

The substrate could facilitate microbial polymorph selection through its specific surface chemistry, surface electrostatic interaction properties, and surface roughness^22^. In this study, the substrate’s physical features, such as roughness and porosity, have not been investigated. To negate their influence, the surface of the substrates was mechanically polished before being mounted into resins. With zeta potential values (magnitude and charge), the strength of repulsion/attraction of charged particles (or molecules) in the system can be interpreted. A lower magnitude of zeta potential (such as in C) indicates that the system is more likely to coalesce, while higher zeta potential values (in magnitude) indicate a stable system that is less likely to agglomerate. Substrates with a high negative zeta potential magnitude, such as A and Q, can attract and hold charged counterions from the solution (e.g., Ca^2+^), creating localised regions of ion accumulation and supersaturation, possibly influencing heterogeneous nucleation (i.e., ionotropic effect)^78^. Moreover, the surface charge indicates how the bacteria and metabolites, including EPS, will attach to the surface of the substrate. At alkaline pH (7.5 to 9.5), the bacterial cells have a net negative zeta potential (~ −40 mV)^79,80^. The bacteria exhibit an electrical double layer that immobilises the Ca^2+^ ions (forming a Stern layer) at the peripheral surface of the microbes^79^. Therefore, the attachment of the negatively charged bacterial cell- Ca^2+^ interface on the apatite and quartz substrates is likely to be weaker than calcite^22,80^. This is possibly a reason for elongated facets/lens shape CaCO_3_ crystals on the calcite substrate. Nonetheless, low-EPS producing SP produces rhombohedral calcite as the bacterial cell acts as a nucleation site, entombing inside the crystals^71^. Previous studies on strain-specific biomineralisation have shown that variations in urease gene expression among bacteria, including SP (formerly *Bacillus pasteurii*), result in elevated urease activity that promotes calcite nucleation and growth near the cell surface under enzyme-induced high pH conditions^21^. The influence of this microbe on polymorph selection is found to be dominant over the surface characteristics of the substrates, in agreement with the findings of Burdalski et al.^35^. Burdalski et al.^35^demonstrated that with various soil minerals substrates, including sand (quartz) and clay minerals (kaolinite, montmorillonite, microcline and forsterite), the predominant precipitation with SP was calcite. These substrate minerals also have substantial differences in their surface electrostatic characteristics^60,81,82^. In the case of BS, the spheroid and hexagonal precipitates were identified as vaterite regardless of the substrate, possibly due to high organic content from the biofilm/EPS, further suggesting that the microbial influence in polymorph selection is dominant in the provided experimental conditions in comparison to the substrate characteristics.

### Influence of microbial species, biofilm and EPS (organics)

This study delivers direct experimental evidence illustrating that biofilm and EPS influence polymorph selection during biogenic CaCO_3_ precipitation. SP produced a relatively low quantity of biofilm and EPS compared to BS, as shown in Fig. 3. Prior studies have reported that biofilms form a globular structure and act as nucleation sites for SP^31^. These studies also revealed that dense biofilms are developed in high nutrient conditions, leading to faster nucleation and smaller crystal sizes^31,37^. In this study, we found SP produces relatively smaller crystals than BS, possibly due to significantly higher urease activity. Prior reports have highlighted that higher urease activity results in smaller CaCO_3_ crystals with low nano-indentation moduli characteristics, while low urease-producing microbes generate larger crystals with higher strength^83^. The influence of microbial species on the polymorph selection of bacterial CaCO_3_ crystals became evident with the microscopic observations of BS-produced precipitates, which are controlled by the biofilm matrix and its functional interaction with the calcium ions on the natural mineral substrates. It is to be noted that the enzymatic activity in BS influences the rate of carbonate production but does not directly determine polymorph selection; instead, they create local heterogeneous supersaturation conditions that favour vaterite formation and stabilisation, particularly under EPS-rich, heterogeneous environments.

EPS can stabilise vaterite by adsorbing calcium ions onto its surface, as demonstrated in this study, and inhibiting its transformation into calcite, which is consistent with Ostwald’s rule of stages. Rodriguez-Navarro et al. (2007) demonstrated that EPS components, such as proteins and amino sugars, are incorporated into vaterite, delaying its through-solution recrystallisation^38^. These organic interactions promote kinetic trapping of the metastable phase. Ostwald’s rule of stages suggests that during the crystallisation process, a system tends to form the least stable phase first, typically the one with the highest solubility^84^. As time progresses, this phase gradually transforms into more stable phases with lower solubility, following an order of increasing stability. The crystallisation sequence for calcium carbonate proceeds as follows: amorphous calcium carbonate (ACC) forms first, followed by vaterite, then aragonite at temperatures above 30–35 °C, and finally calcite^22,29^. This sequential transformation is driven by the thermodynamic tendency of the system to move toward a lower energy state. In the case of the low-EPS route with SP, the reaction kinetics are not hindered by organics like EPS, allowing rapid reaction rates that lower the energy barrier and facilitate final calcite formation^85^.

Li et al. (2021) also demonstrated that biofilm-EPS can accumulate cations, and mineralisation starts at the base of the biofilm^32^. The bacterial cells also localise for their metabolic activities, creating localised supersaturation near EPS due to the availability of moisture and micronutrients^3,9,45^. Moreover, previous studies using SEM and High-Resolution Transmission Electron Microscopy (HRTEM) have shown that hexagonal, lens-, and rosetta-shaped CaCO_3_ forms are driven by ammonium-stabilised vaterite nanosheets assembling into single crystal structures (i.e., mesocrystals) and crystallising after amorphous nanoparticles, with the EPS-ammonium interaction influencing their lens shape^86^ Another study demonstrated that the hexagonal plate-like vaterite formation is promoted in the presence of NH_4_^+^ ions and is often preceded by an amorphous precursor phase^73^. The polymeric EPS network is likely to retain the ammonium ions, and possibly is the reason for the formation of the pseudo-hexagonal vaterite structures observed here. In this study, dominant vaterite formation occurs in the medium that contains bacterial cells, metabolic organic constituents, ammonia/ammonium ions (produced through hydrolysis of urea) and EPS.

Vaterite crystals are commonly reported to have a hexagonal structure, although the exact crystal system and space group are a matter of discussion^86–90^. Recent studies have demonstrated that vaterite crystals commonly display a pseudo-hexagonal mesostructure with a pseudo-sixfold axis along the [103]_m_ direction (for the monoclinic structure)^91^. Importantly, the crystals showed corrugated and hollow areas with a worm- or maze-like structure, likely resulting from the entrapment of organics (e.g., EPS) during vaterite precipitation. The processes of random heterogeneous nucleation on EPS, crystal growth orientation, and the transformation of metastable phases during biomineralisation can influence the shape of the precipitated calcium carbonate crystals^39^. Similarly, organic (macro)molecules such as polysaccharides (a type of carbohydrate) have been reported to have aggregating properties^92^. In the case of EPS, the location of available entrapped Ca^2+^ also depends on the orientation and composition of the functional groups of EPS, determining which (*hkl*) plane of the newly-formed (CaCO_3_) phase will preferentially interact with the substrate or even which polymorph will crystallise^93^.

In-depth investigations were conducted with advanced tools such as ToF-SIMS and FTIR to understand the underlying mechanism of polymorph selection with EPS. The total carbohydrate and protein content in the EPS produced by SP and BS was also investigated. It has been discovered that the organics (carbohydrates) present in the EPS matrix play a crucial role in entrapping calcium ions and vaterite stabilisation. The adsorbed calcium ions on the organic matrix can subsequently interact with carbonate ions, resulting in the precipitation of calcium carbonate, as observed during the precipitation of CaCO_3_ via ion binding in the presence of a biomimetic organic matrix^94^.

The composition of EPS and FTIR spectra provided key molecular insights into the functional groups of organics that are likely responsible for trapping calcium ions within the matrix and promoting vaterite biomineralisation through BS activity. It is well known that the selection of CaCO_3_ polymorphs is influenced by variations in the biochemical composition of EPS extracted from different stromatolite layers^95,96^. Most of the studies on cyanobacterial calcification processes have emphasised the role of proteins, carboxylate groups and amino acids in the bacterial mineralisation process^90,97^. This is, however, the first study that explores the role of EPS along with the urea hydrolysis pathway on bacterial mineralisation and polymorph selection. EPS typically includes high amounts of carbohydrates, reaching concentrations of up to ~ 700 mg/g^75^. In this case, FTIR analysis revealed that the saturated carbohydrates, amino acids (-NH_2_), hydroxyl (–OH), carboxyl (–COOH) and complex carbohydrate skeleton rings might be the keys that influence the morphology and mineralogy of microbial crystals. Previous studies have emphasised the role of different EPS macromolecules on polymorph selection in biogenic crystal precipitation systems^3,38,42,97–99^. It is reported that vaterite production is facilitated by amino acids (specifically aspartic and glutamic acids)^97–99^. Moreover, vaterite production is known to be favoured in the presence of low-molecular-weight organics, amides, carboxylic groups and bacterial metabolites^38,87,88,97,98,100^. Carboxylate (COO⁻) groups are the most prevalent and effective functional groups for binding cations during biomineral nucleation^101^. Hydroxyl groups can influence biomineralisation by forming hydrogen bonds and coordinating with metal ions, thereby affecting nucleation and crystal growth. These functional groups are likely involved in binding calcium ions and nucleating mineral phases, thereby influencing the size, distribution, and morphology of the precipitates. In contrast, Tourney et al. reported that EPS (extracted/cell-free) inhibits vaterite formation owing to the dissolved organic carbon released from EPS through an ammonia-free drift method^3^. In the current study, however, a ureolytic microbial pathway of calcium carbonate precipitation was employed that is liable to release ammonium ions in the system^1,102^. On the other hand, it has also been reported that in the presence of enough unutilised calcium and urea in the system, bacterial precipitation favours vaterite stabilisation^39^. In the case of BS in the current study, the carbohydrates and proteins present in the EPS act as crystallisation inhibitors by binding Ca²⁺ in solution and delaying nucleation, which allows high supersaturation to build and favours vaterite formation^38^. These biomolecules also serve as nucleation templates and become embedded within or adsorbed onto forming crystals, thereby stabilising vaterite against transformation into more stable polymorphs^38,64,103^.

Although the EPS produced by SP and BS had no significant variations in the profiles of the functional groups, the quantity of EPS produced by SP was significantly lower than that produced by BS. Moreover, BS has higher carbohydrate content and lower protein content than SP. Combined analyses using Raman, FTIR, and EPS characterisation revealed differences in polymorph selection associated with varying EPS abundance, while ToF-SIMS demonstrated the adherence of organic compounds (carbohydrates) to the substrate and associated precipitates. These findings indicate that carbohydrates within the biofilm (cells and EPS) matrix play a critical role in controlling the size, phase, and morphology of CaCO₃ precipitates.

The composition of EPS and its functional groups requires further investigation to identify which specific group affects the selection of polymorphs, size and shape of the resulting precipitates. It is to be noted that the findings of the current study are confined to the particular ureolytic bacterial strains, composition and concentration of cementation medium, and environmental conditions (pH and *T*) tested here. Only three natural substrates were examined without considering the influence of physical features such as pores and surface roughness. Moreover, the relatively small quantity of bacterial precipitates made it challenging to identify their mineralogy. Although Raman and ToF-SIMS analyses were instrumental in the current study to unravel the mineral phases and their interaction with the bacterially produced organics, i.e., EPS, other advanced mineralogical and microscopy tools such as Atomic Force Microscopy (AFM), HRTEM and Nanoscale Secondary Ion Mass Spectrometry (Nano-SIMS), among others, could help in disclosing further details of the interactions between the natural substrates, organics and bacterial biominerals.

## Conclusion

This study offers a fundamental understanding of the mechanisms by which bacterially derived organic compounds control the shape, size, and phase of biogenic CaCO_3_. This study determined that the microbial route and the presence of organic content, particularly in the form of biofilm and EPS, have a dominant influence on polymorph selection and bacterial mineral morphology as compared to substrate mineralogy and electrostatic interactions. The biominerals formed in the low-EPS route were observed to be predominantly rhombohedral calcite crystals regardless of the substrate, whereas, in the high-EPS producing route, spheroid and pseudo-hexagonal vaterite crystals were observed. The crystal growth mode in EPS-derived minerals was significantly different from that in the low-EPS route. In the low-EPS route, the well-known rhombohedral morphology of calcite was observed with epitaxial growth. In contrast, the EPS route involves hosting microbial cells, entrapping calcium ions, and acting as a binding agent for precipitated bacterial minerals. Using advanced surface-analytical techniques (ToF-SIMS) along with molecular fingerprinting with FTIR and mineralogical phase confirmation with Raman spectroscopy, this study shows that the organics (carbohydrate and carboxylate functional groups) adhere to the substrates, bind calcium ions and play a critical role in the formation and stabilisation of vaterite structures.

Although this study has revealed crucial findings regarding the role of organics in the nucleation and crystal growth of bacterial biominerals, further investigations are required to understand natural systems. Future studies should consider factors such as biofilm adhesion to different substrates, substrate surface characteristics (e.g., porosity), and the influence of pH, humidity, and *P-T* conditions on EPS’s ability to entrap calcium ions and foster CaCO_3_ biomineralisation. For calcium-based substrates, an isotope-based (Ca-44) study is recommended to differentiate between biogenic and abiogenic minerals. Nevertheless, a strategic investigation of various pure, natural, and complex engineered substrates is necessary to better understand bacterial mineralisation processes in different environmental settings.

## Supplementary Information

Below is the link to the electronic supplementary material.

Supplementary Material 1

## Data Availability

All data generated or analysed during this study are included in this article.
