# Supplementary material for: Effect of substrate mineralogy, biofilm and extracellular polymeric substances on bacterially induced carbonate mineralisation investigated with in situ nanoscale ToF-SIMS
Source: Sci Rep. 2025 Aug 11;15:29368. doi: 10.1038/s41598-025-14083-z (PMC12339710; doi:10.1038/s41598-025-14083-z)
Supplement: Supplementary file 1 — Supplementary Material 1 [file 41598_2025_14083_MOESM1_ESM.pdf]

<u>**Supplementary Figures**</u>

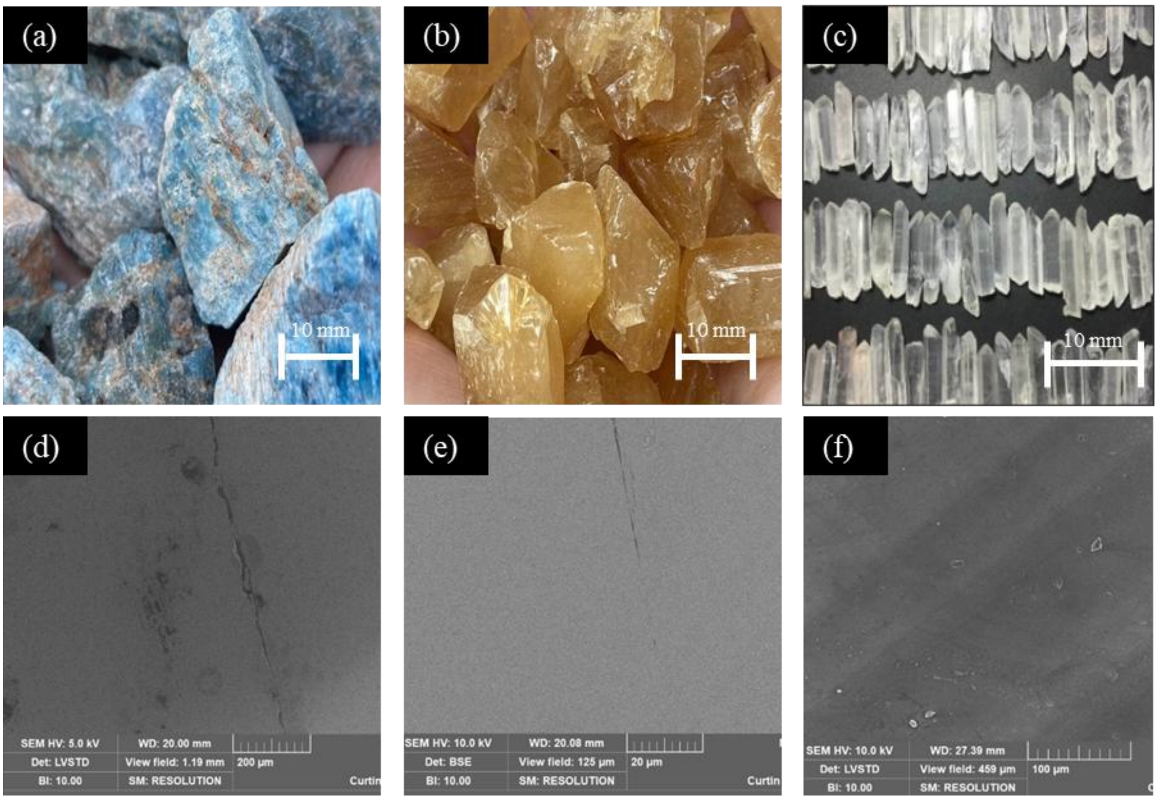

Fig. S1 Photos of substrates: (a) Apatite; (b) Calcite; (c) Quartz; and SEM photomicrographs showing surface features of the substrates: (d) Apatite; (e) Calcite; and (f) Quartz at the microscale.

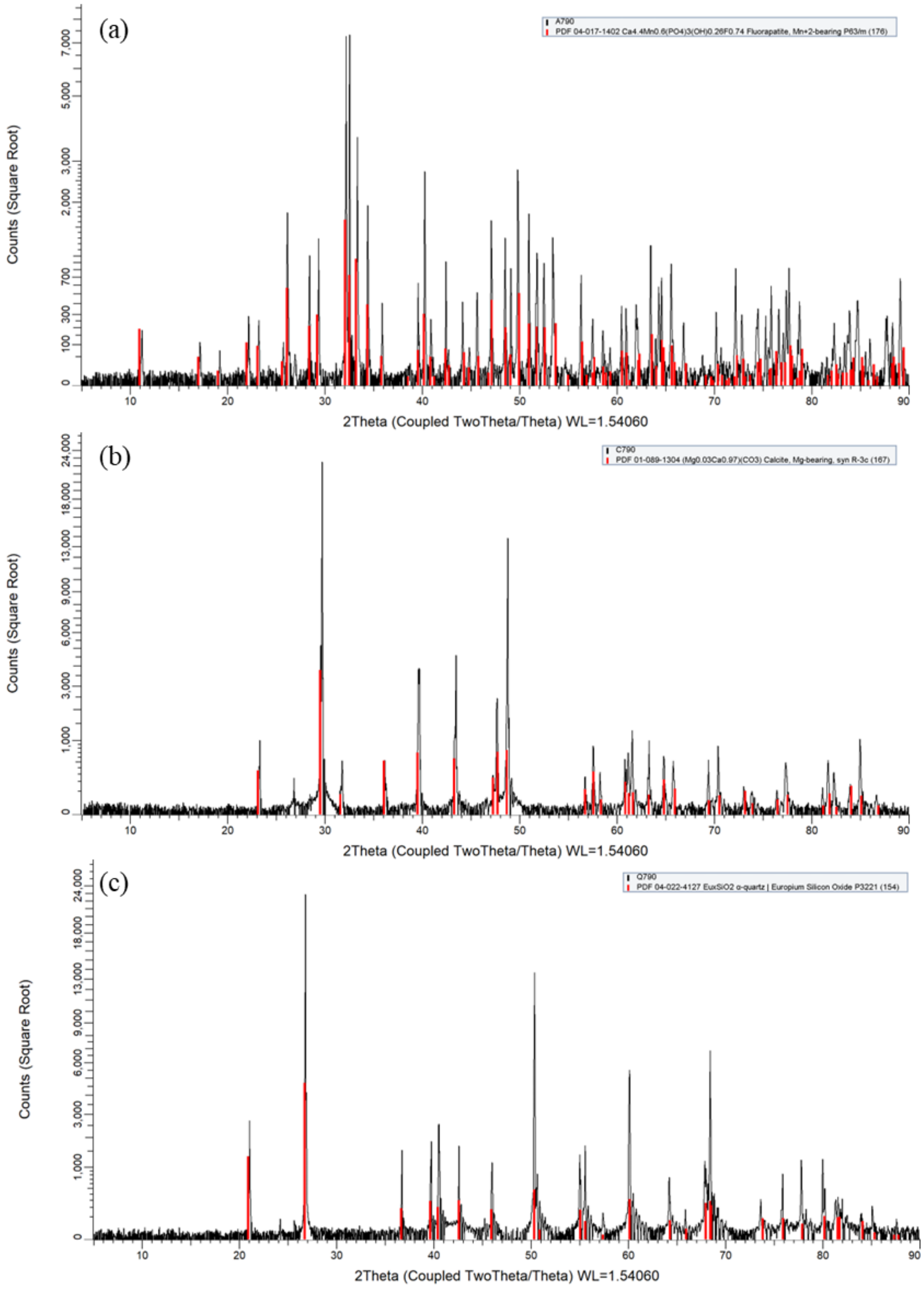

Fig. S2 Mineralogy of the substrates determined by XRD: (a) apatite, (b) calcite, and (c) quartz.

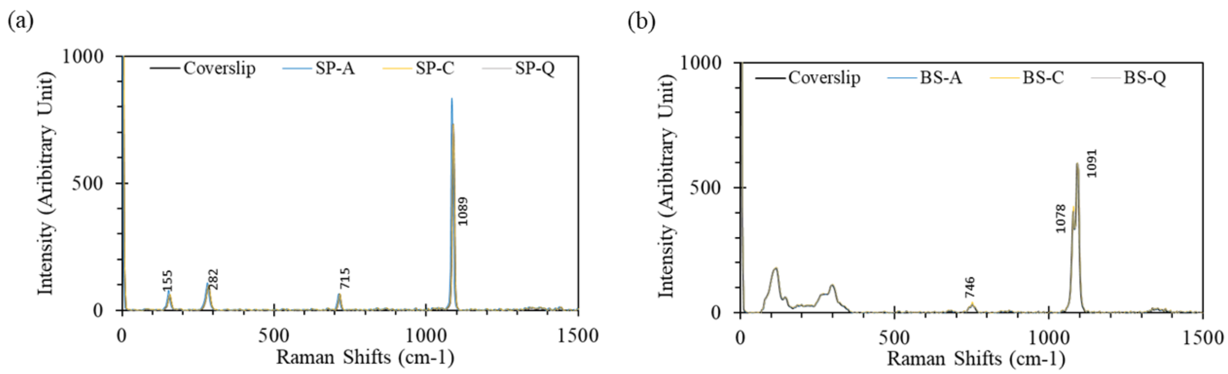

Fig. S3 Mineralogical analysis of the bacterial precipitate with one cycle of treatment with Raman spectroscopy. (a) Raman spectrum showing the matching principal bands of calcite crystals formed by SP on coverslip, apatite (SP-A), calcite (SP-C) and quartz (SP-Q); and (b) Raman spectrum showing the matching principal bands of vaterite crystals formed by BS on coverslip, apatite (BS-A), calcite (BS-C) and quartz (BS-Q).
